# Impact of Selective Renal Afferent Denervation on Oxidative Stress and Vascular Remodeling in Spontaneously Hypertensive Rats

**DOI:** 10.3390/antiox11051003

**Published:** 2022-05-20

**Authors:** Lu-Lu Wu, Yue Zhang, Xiu-Zhen Li, Xin-Li Du, Ying Gao, Jing-Xiao Wang, Xiao-Li Wang, Qi Chen, Yue-Hua Li, Guo-Qing Zhu, Xiao Tan

**Affiliations:** 1 Key Laboratory of Targeted Intervention of Cardiovascular Disease, Collaborative Innovation Center of Translational Medicine for Cardiovascular Disease, and Department of Physiology, Nanjing Medical University, Nanjing 211166, China; luluuu@njmu.edu.cn (L.-L.W.); wangjx@njmu.edu.cn (J.-X.W.); xiaoliwang@njmu.edu.cn (X.-L.W.); 2 Emergency Department, The Second Affiliated Hospital of Nanjing Medical University, Nanjing 211166, China; zhangyueefy@njmu.edu.cn (Y.Z.); lixiuzhen0806@njmu.edu.cn (X.-Z.L.); xinliheihei@njmu.edu.cn (X.-L.D.); nydgaoying@njmu.edu.cn (Y.G.); 3 Department of Pathophysiology, Nanjing Medical University, Nanjing 211166, China; qichen@njmu.edu.cn (Q.C.); yhli@njmu.edu.cn (Y.-H.L.)

**Keywords:** oxidative stress, renal afferents, hypertension, sympathetic activity, vascular remodeling

## Abstract

Oxidative stress and sustained sympathetic over-activity contribute to the pathogenesis of hypertension. Catheter-based renal denervation has been used as a strategy for treatment of resistant hypertension, which interrupts both afferent and efferent renal fibers. However, it is unknown whether selective renal afferent denervation (RAD) may play beneficial roles in attenuating oxidative stress and sympathetic activity in hypertension. This study investigated the impact of selective RAD on hypertension and vascular remodeling. Nine-week-old normotensive Wistar-Kyoto rats (WKY) and spontaneously hypertensive rats (SHR) were subjected to selective renal afferent denervation (RAD) with 33 mM of capsaicin for 15 min. Treatment with the vehicle of capsaicin was used as a control. The selective denervation was confirmed by the reduced calcitonin gene-related peptide expression and the undamaged renal sympathetic nerve activity response to the stimulation of adipose white tissue. Selective RAD reduced plasma norepinephrine levels, improved heart rate variability (HRV) and attenuated hypertension in SHR.It reduced NADPH oxidase (NOX) expression and activity, and superoxide production in the hypothalamic paraventricular nucleus (PVN), aorta and mesenteric artery of SHR. Moreover, the selective RAD attenuated the vascular remodeling of the aorta and mesenteric artery of SHR. These results indicate that selective removal of renal afferents attenuates sympathetic activity, oxidative stress, vascular remodeling and hypertension in SHR. The attenuated superoxide signaling in the PVN is involved in the attenuation of sympathetic activity in SHR, and the reduced sympathetic activity at least partially contributes to the attenuation of vascular oxidative stress and remodeling in the arteries of hypertensive rats.

## 1. Introduction

Oxidative stress is characterized by either excess oxidants or low antioxidants [1]. One of the major sources of reactive oxygen species (ROS) is a family of NADPH oxidases (NOXs) including NOX1, NOX2 and NOX4 in vasculature [2,3,4]. Superoxide may serve as cell signaling in pathophysiological processes [5], but excessive ROS production leads to protein oxidation, cell signaling disturbance, proliferation, migration, fibrosis, inflammation and apoptosis, which are important pathological processes for the vascular remodeling and dysfunction in hypertension [6,7,8]. More than that, oxidative stress in the hypothalamic paraventricular nucleus (PVN) causes sympathetic over-activation [9], and persistently excessive sympathetic activity contributes to hypertension and vascular remodeling [10]. Vascular remodeling plays a crucial role in the development of hypertension and related complications [11].

Sympathetic over-activation is crucial for the development of hypertension and organ damage [12]. Inhibition of sympathetic activity is a strategy for preventing hypertension and related complications [13] There is a close relationship between kidney and sympathetic hyperactivity in chronic kidney diseases and hypertension [14,15]. We have found that stimulation of renal afferents with capsaicin induces excitatory renal reflex (ERR), leading to increases in sympathetic activity and blood pressure in normal rats [16]. The ERR is mediated by angiotensin II and its AT_1_ receptors in the PVN. Then, the activated AT_1_ receptors promote NOX-dependent superoxide production [17] and subsequent IL-1β production in the PVN [18]. The renal afferent activity increases the activity of some PVN neurons [19], and PVN damage prevented the capsaicin-induced ERR, suggesting that the PVN is an important integrating center of the ERR [16]. More recently, we have showed that the ERR in spontaneously hypertensive rats (SHR) was enhanced at the early stage of hypertension, but attenuated at the later stage of hypertension compared with Wistar-Kyoto rats (WKY), suggesting that renal afferent activity contributes to excessive sympathetic activation in SHR, especially in the early stage of hypertension [20].

Renal nerves contain both afferent fibers and efferent fibers. The input from renal afferents to the brain causes sympathetic activation pressor responses [21,22]. Catheter-based renal denervation is widely used as a strategy for intervention of resistant hypertension, and the surgery destroys both afferent and efferent renal fibers, which may cause great side effects [23,24]. Recently, it has been found that unilateral selective renal afferent denervation (RAD) attenuates sympathetic activity and renal dysfunction in the rats with ipsilateral ischemic kidney [25], and in the rats with unilateral 5/6 nephrectomy [26]. However, the effects of RAD were only investigated in the rat model of chronic kidney disease without the studies in essential hypertension. It is well known that essential hypertension accounts for more than 95% of the total patients with hypertension. It is still unknown whether RAD could play beneficial roles in attenuating oxidative stress, vascular remodeling and hypertension. SHR are the most commonly used rat model of essential hypertension. The hereditary hypertension model has similarities with human essential hypertension in terms of pathophysiological process, neuroendocrine changes, clinical course and complications [27,28]. This study is designed to investigate the effects of renal afferent denervation on central and vascular oxidative stress, vascular remodeling and hypertension in SHR.

## 2. Materials and Methods

### 2.1. Animals

Male WKY and SHR were obtained from the Vital River Laboratory Animal Technology Co., Ltd. (Beijing, China). The experimental intervention was applied to the rats aged 9 weeks. The study was conducted in accordance with the NIH guidelines, and approved by the Experimental Animal Care and Use Committee of Nanjing Medical University (No. 1811017-1, 16 October 2020). The criterion for the rats used in this study was that the systolic blood pressure (SBP) of SHR was higher than 150 mmHg and that of WKY was lower than 140 mmHg. One SHR’s SBP was lower than this standard and was excluded from the experiment. The rats were housed in standard polypropylene cages in a temperature- and humidity-controlled room with a 12/12 h light/darkness cycle, and were allowed free access to tap water and normal chow. Rats were euthanized by intravenous injection of pentobarbital sodium (150 mg/kg).

### 2.2. Renal Afferent Denervation

Either WKY or SHR were randomly divided into the RAD group and the control group, and were anaesthetized with sodium pentobarbitone (50 mg/kg). The RAD surgery was performed according to the previous report with a little modification [25,26,29]. There are some renal nerve fibers or thin renal nerves walking along the renal artery and vein in addition to obvious renal nerves. In order to abolish all the renal afferent activity, capsaicin was applied to the renal nerves, artery and vein. Simply, the rats were kept prone, and surgery was performed with sterile techniques. Bilateral kidneys were exposed retroperitoneally via flank incision. The renal artery, vein and nerve were gently isolated from the connective tissue without damage to the renal nerve. After placing a small piece of parafilm under the renal artery and vein to prevent the capsaicin exposure to other tissues, a small piece of gauze soaked in 33 mM of capsaicin solution was wrapped around the renal artery and vein. Visible renal nerves were painted with 33 mM of capsaicin (MedChem Express, Monmouth Junction, NJ, USA) solution. Following 15 min of capsaicin exposure, the gauze and parafilm were removed, and the incision was sutured. The capsaicin was dissolved in the vehicle composed of 90% normal saline, 5% ethanol, and 5% Tween 80 for this study. In the control rat, the procedure was similar to that of the RAD rat, except that the vehicle of capsaicin was used instead of capsaicin solution. The blood pressure of the tail artery was measured every week, and all other measurements were performed 3 weeks after the surgery.

### 2.3. Identification of RAD

RAD reliability was identified by reduced afferent sensory innervation and intact efferent sympathetic innervation for the kidney. Calcitonin gene-related peptide (CGRP) protein expression was used as a marker for detecting afferent innervation [30,31]. Chemical stimulation of white adipose tissue (WAT) elicited sympathetic excitation and pressor responses called adipose afferent reflex (AAR) [32]. The AAR was evaluated, and renal sympathetic nerve activity (RSNA) was recorded as we previously reported [33,34]. The right inguinal WAT was exposed via an inguinal area incision. Four stainless steel tubes (0.31 mm outer diameter) were inserted into the WAT 3 mm deep below the surface. The tubes were 4 mm apart from each other and connected to a 4-channel programmable pressure injector (PM2000B, MicroData Instrument, NJ, USA). Capsaicin (1.0 nmol/μL) was simultaneously infused into the four sites of the inguinal WAT at a rate of 4.0 μL/min for 2 min to induce the AAR. The left RSNA response to inguinal WAT stimulation with capsaicin was used as a parameter to evaluate the undamaged sympathetic response. The RSNA was amplified with an AC/DC differential amplifier (model 3000; A-M system in Washington, DC, USA), filtered with a band-pass of 60 to 3000 Hz, and integrated at a time constant of 100 ms. The signals were recorded with a Powerlab Data Acquisition System (8/35, ADInstruments, Sydney, NSW, Australia). The RSNA response was expressed as a percentage change from the baseline.

### 2.4. Measurement of Blood Pressure and Heart Rate

The blood pressure and heart rate of the tail artery were examined with a noninvasive computerized tail-cuff system and PowerLab system with data acquisition software (ADInstruments, Bella Vista, NSW, Australia) in a conscious state as we previously reported [20]. Before the experiments, the blood pressure of the tail artery was measured in three consecutive days to make them accustomed to the environment and the cuff measurement. The data was obtained by averaging 10 measurements.

### 2.5. Examination of Plasma Norepinephrine Level and Heart Rate Variability

Both plasma norepinephrine (NE) level and heart rate variability (HRV) were used as indices of sympathetic activity. Plasma NE level was measured with Commercial ELISA kits (R&D systems, Minneapolis, MN, USA) according to the manufacturer’s descriptions. HRV was measured as we recently reported [35]. Simply, HRV analysis was performed according to an electrocardiograph (ECG) recorded with subcutaneous electrodes of standard II configuration. HRV was determined by frequency domain as follows: low-frequency (LF) power: 0.20 to 0.75 Hz; high-frequency (HF) power: 0.75 to 2.50 Hz; very-low-frequency power (VLF): 0 to 0.20 Hz; total power (TP): 0 to 3.00 Hz. Normalized low frequency (nLF) is an index of sympathetic activity, and normalized high frequency (nHF) serves as an index of parasympathetic activity. The ratio of nLF to nHF represents the sympathetic–parasympathetic balance [36,37]. The following equations were used to calculate nLF and nHF: nLF = 100 × LF/(TP-VLF); nHF = 100 × HF/(TP-VLF). The values are expressed as normalized units (nu).

### 2.6. Measurement of NOX Activity and Superoxide Production

NOX activity and superoxide production in the PVN, aorta and mesenteric artery were examined [35,38]. Tissue was quickly frozen in liquid nitrogen, and measurements were performed within 3 days. The tissues in lysis buffer were homogenized and centrifuged. The total protein in the supernatant was measured with the Bradford assay (BCA; Pierce, Santa Cruz, CA, USA). NOX activity and superoxide level in the supernatant were measured with the lucigenin-derived chemiluminescence method. A modified HEPES buffer was used in the measurements. Photon emission was triggered by both dark-adapted lucigenin (5 μM) and NAD(P)H (100 μM) for measuring NOX activity. Photon emission was initiated by administration of dark-adapted lucigenin (5 μM) for determining superoxide anion production. Values were obtained by averaging ten measurements in 10 min with a luminometer (Model 20/20 n, Turner, CA, USA). Background chemiluminescence was determined in the buffer containing lucigenin (5 μM). Values were expressed as mean light unit (MLU)/min/mg protein.

### 2.7. In Situ Detection of Superoxide Anions in the PVN

Specific fluorogenic probe dihydroethidium (DHE) was used to show the superoxide production in the PVN as we reported previously [35,38]. Samples from all groups were processed in parallel. Rats were euthanized with an overdose of pentobarbital sodium (150 mg/kg). Brains were quickly removed, frozen in liquid nitrogen and embedded in tissue OCT-freeze medium. Then, coronal sections at 30 μm of thickness were made. The sections were thawed at room temperature, rehydrated with phosphate-buffered saline and treated with DHE (1 μmol/L) in the dark for 5 min. After washing with phosphate-buffered saline, the DHE fluorescence was detected by a fluorescence microscope (BX51, Olympus, Tokyo, Japan) using an excitation wavelength of 543 nm and a rhodamine emission filter. Detector and laser settings were kept constant for all samples. DHE fluorescence in sections was examined with a fluorescence microscope (BX51, Olympus, Tokyo, Japan) using an excitation wavelength of 543 nm and a rhodamine emission filter. The images were analyzed with the software of Image Pro Plus 6.0 (Media Cybernetics, Silver Spring, MD, USA).

### 2.8. Masson’s Staining

Tissue was fixed with paraformaldehyde. The paraffin-embedded aorta or mesenteric artery was sectioned and stained with Masson’s trichrome staining with standard protocols. The images were taken under a light microscope. Media thickness, lumen diameter and the ratio of media thickness to lumen diameter were analyzed with the software of Image Pro Plus 6.0 and used to evaluate vascular remodeling [39,40].

### 2.9. Western Blot and Antibodies

Protein extracts from samples were separated on 10% SDS-PAGE and transferred onto PVDF membrane. The membrane was blocked with 5% non-fat milk in TBST and then probed with primary antibody (1:1000) at 4 °C overnight followed by incubation with HRP-linked secondary antibody (1:5000) [41]. NOX1, NOX2, NOX4 and β-actin antibodies were obtained from Proteintech Group Inc. (Rosemont, IL, USA). CGRP antibodies were bought from Abcam (Cambridge, MA, USA).

### 2.10. Examination of Aldosterone, Potassium and Sodium Levels in Serum and Urine

Serum and urine aldosterone levels were measured with commercial ELISA kits (E-EL-0070c, Elabscience, Wuhan, Hubei, China) according to the manufacturer’s descriptions. The colorimetric method was used for analysis of potassium and sodium. Serum and urine potassium and sodium levels were, respectively, measured with a commercial Potassium Assay kit and Sodium Assay kit (C003-1-1 and C002-1-1, Jiancheng Bioengineering Institute, Nanjing, Jiangsu, China) following the manufacturer’s descriptions.

### 2.11. Statistics

The experiment was conducted in a randomized double-blind manner. The number of each group represented the number of samples from different rats. All statistical analyses were done using computer software (SigmaStat, SPSS22.0, Chicago, IL, USA). Data were expressed in mean ± SE. One-way or two-way ANOVA followed by Bonferroni’s post hoc analysis were used for comparisons. The distribution of all data was examined before analysis of variance. All data in this study are normally distributed. *p* < 0.05 was considered statistically significant.

## 3. Results

### 3.1. Identification of the RAD Reliability

**Figure 1:**
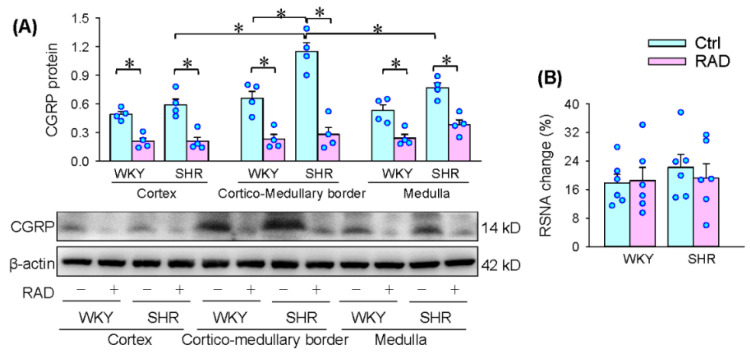

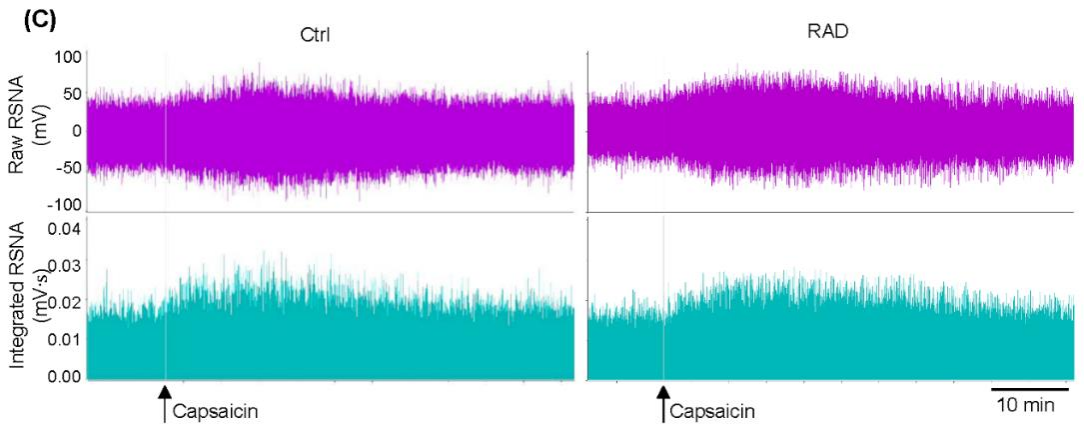
Identification of renal afferent denervation (RAD) in WKY and SHR. (**A**) CGRP protein expression in the cortex, cortico-medullary border and medulla of the kidney, which is used as a marker of afferent innervation of the kidney. (**B**) RSNA response to the infusion of capsaicin to the inguinal white adipose tissue, which is used to confirm the intact renal efferent activity. (**C**) Representative recordings showing the RSNA response to the infusion of capsaicin to the inguinal white adipose tissue in SHR. Values are mean ± SE. Two-way ANOVA followed by Bonferroni’s post hoc analysis were used for comparisons. * *p* < 0.05. *n* = 4 for each group in (**A**). *n* = 6 for each group in (**B**).

The CGRP protein serves as a marker of sensory innervation [30,31]. RAD reduced the CGRP protein expression in the cortex, cortico-medullary border and medulla of the kidney, suggesting that RAD effectively reduced renal afferent innervation (Figure 1A). Stimulation of WAT with capsaicin increased the RSNA and pressor responses called AAR [32]. We examined the sympathetic response to inguinal WAT stimulation with capsaicin, which was used as the index of the undamaged function of renal efferent sympathetic activity. The AAR was induced as we previously reported [33,34]. There were no significant differences in the RSNA response to capsaicin between the Ctrl and the RAD of WKY and SHR (Figure 1B). The duration and pattern of the RSNA response to capsaicin in the RAD-treated rat was similar to the control rat (Figure 1C).

### 3.2. Blood Pressure and Heart Rate

**Figure 2:**
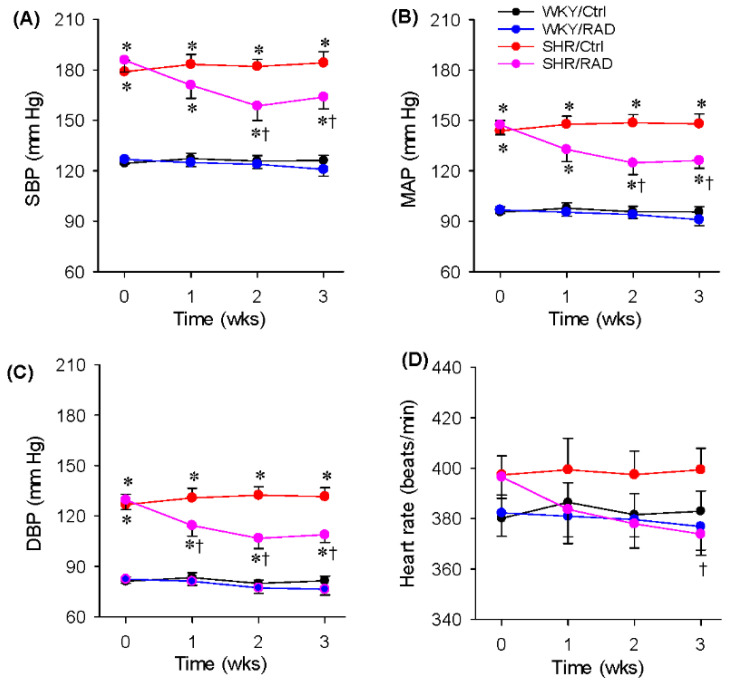
Effects of RAD on the blood pressure of the tail artery in WKY and SHR: (**A**) systolic blood pressure (SBP); (**B**) mean arterial pressure (MAP); (**C**) diastolic blood pressure (DBP); (**D**) heart rate. Values are mean ± SE. One-way ANOVA followed by Bonferroni’s post hoc analysis were used for comparisons. * *p* < 0.05 vs. WKY; † *p* < 0.05 vs. Ctrl. *n* = 6 for each group.

RAD caused a lasting reduction in blood pressure, including systolic blood pressure, mean arterial pressure and diastolic blood pressure in SHR. The maximal depressor response was observed at the third week (about −15%), lasting at least 3 weeks (Figure 2A–C). RAD induced a tendency to reduce heart rate in the first two weeks in SHR, but there was no significant difference. Significantly, reduced heart rate was observed at the 3rd week after RAD in SHR (Figure 2D). Moreover, RAD had no significant effects on blood pressure and heart rate in WKY.

### 3.3. Plasma NE Level and HRV

**Figure 3:**
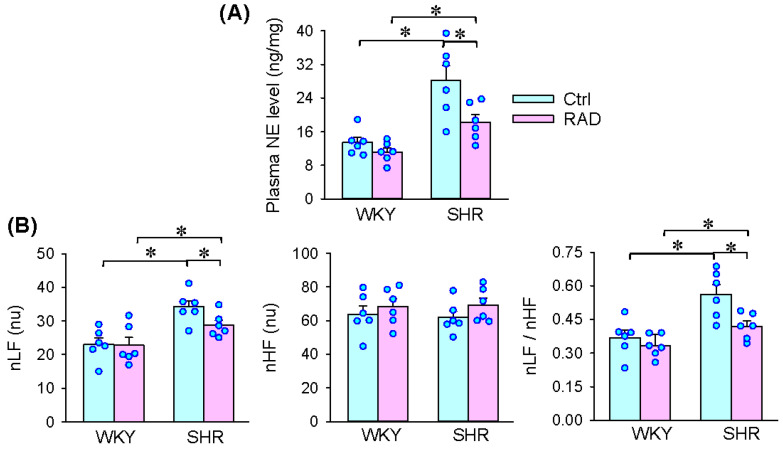
Effects of RAD on the plasma norepinephrine (NE) level and heart rate variability (HRV) in WKY and SHR: (**A**) plasma NE level; (**B**) HRV. nLF, normalized low frequency; nHF, normalized high frequency. Values are mean ± SE. * *p* < 0.05. *n* = 6 for each group.

Excessive sympathetic activity contributes to the pathogenesis of hypertension [12]. The plasma NE level was increased in SHR, which was prevented by RAD (Figure 3A). Moreover, RAD attenuated the increased nLF and the nLF/nHF ratio in SHR, but had no significant effect on nHF (Figure 3B). The findings indicate that abolishing renal afferent activity in SHR attenuates sympathetic activity, which further provides evidence that renal afferent activity is at least partially responsible for the excessive sympathetic activation in SHR.

### 3.4. Superoxide Production in the PVN

**Figure 4:**
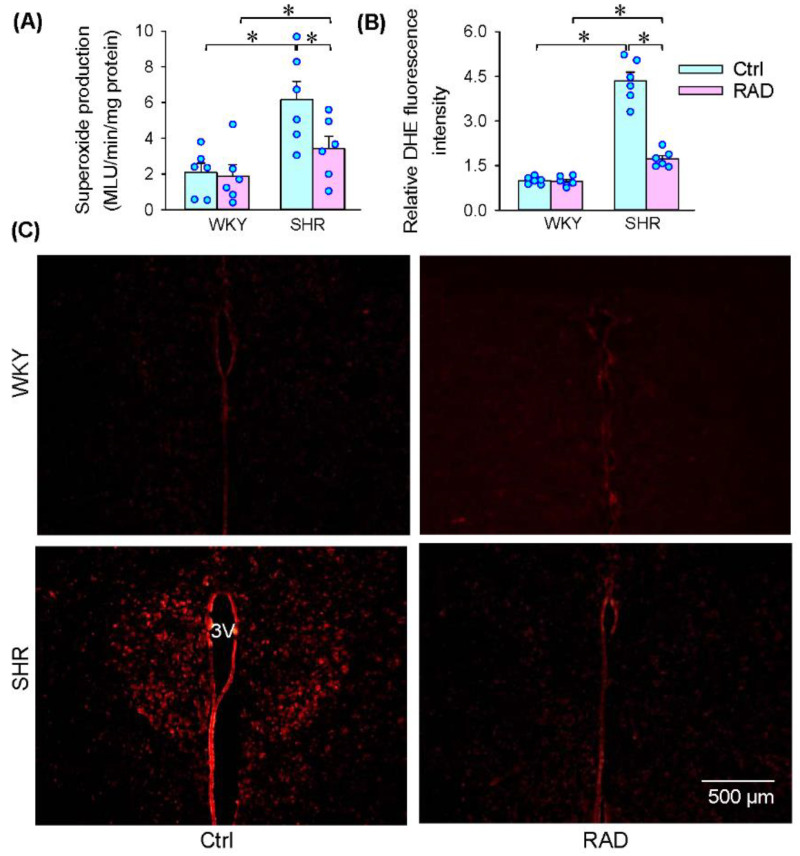
Effects of RAD on superoxide production in the PVN of WKY and SHR. (**A**) Superoxide production. (**B**) Bar graphs showing relative DHE fluorescence intensity. (**C**) Representative images showing DHE fluorescence staining in the PVN areas. 3 V, the third ventricle. Values are mean ± SE. Two-way ANOVA followed by Bonferroni’s post hoc analysis were used for comparisons. * *p* < 0.05. *n* = 6 for each group.

Superoxide signaling in the PVN mediates several excitatory sympathetic reflexes including excitatory renal reflex, and increased superoxide production in the PVN contributes to excessive sympathetic activity in SHR [9,18]. Superoxide production was increased in the PVN of SHR, which was inhibited by RAD (Figure 4A). Similar results were observed in the DHE fluorescence intensity changes (Figure 4B). These findings indicate that RAD attenuates superoxide signaling or oxidative stress in the PVN, which may be responsible for the inhibitory effects of RAD on sympathetic activity. DHE fluorescence staining showed that RAD in SHR reduced the superoxide production in both the magnocellular and parvocellular division of the PVN (Figure 4C).

### 3.5. NOX Activity and Expression in the PVN

**Figure 5:**
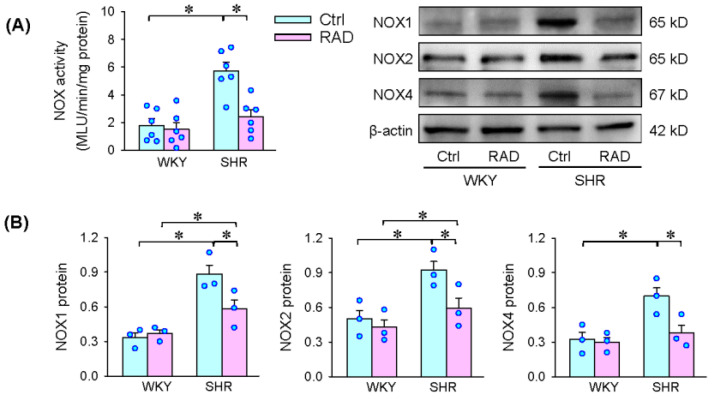
Effects of RAD on NOX activity and expression in the PVN of WKY and SHR. (**A**) NOX activity. (**B**) NOX1, NOX2 and NOX4 protein expressions. Values are mean ± SE. Two-way ANOVA followed by Bonferroni’s post hoc analysis were used for comparisons. * *p* < 0.05. *n* = 6 for each group in A; *n* = 3 for each group in B.

To determine the origin of superoxide production in the PVN, NOX activity and expression were examined. The increased NOX activity was inhibited by RAD in SHR (Figure 5A). The upregulation of NOX1, NOX2 and NOX4 protein expression were attenuated by RAD in SHR (Figure 5B), suggesting that the roles of RAD in reducing superoxide production are attributed to NOX downregulation.

### 3.6. Oxidative Stress in Arteries

**Figure 6:**
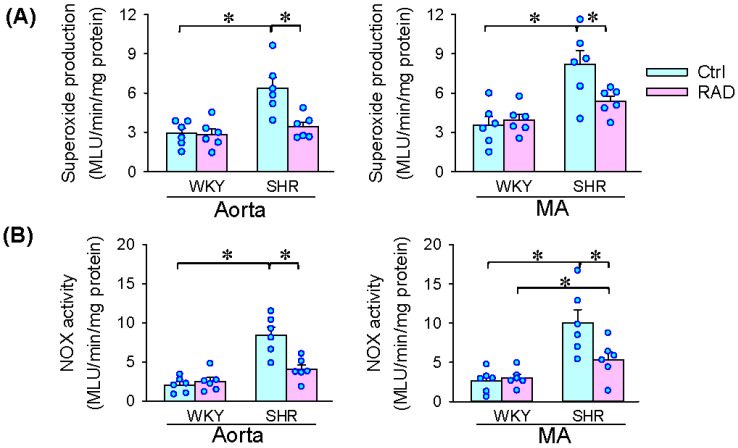
Effects of RAD on superoxide production and NOX activity in the aorta and mesenteric artery of WKY and SHR. (**A**) Superoxide production. (**B**) NOX activity. Values are mean ± SE. Two-way ANOVA followed by Bonferroni’s post hoc analysis were used for comparisons. * *p* < 0.05. *n* = 6 for each.

**Figure 7:**
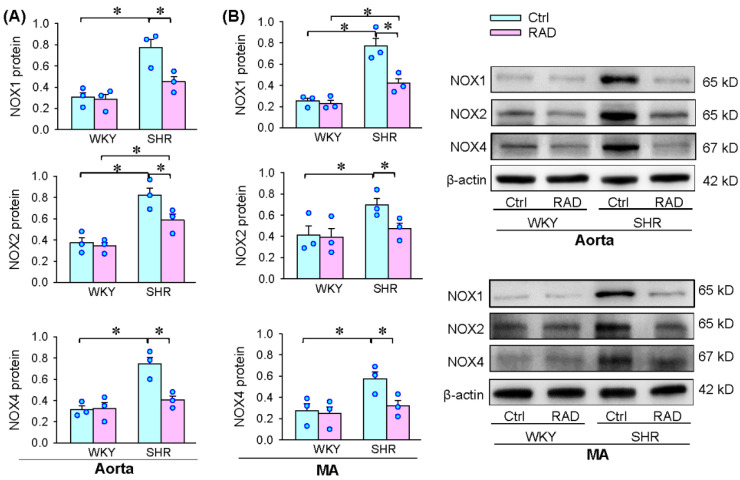
Effects of RAD on NOX protein expressions in the aorta and mesenteric artery (MA) of WKY and SHR. (**A**) NOX1, NOX2 and NOX4 protein expressions in the aorta. (**B**) NOX1, NOX2 and NOX4 protein expressions in the MA. Values are mean ± SE. Two-way ANOVA followed by Bonferroni’s post hoc analysis were used for comparisons. * *p* < 0.05. *n* = 3 for each group.

Vascular oxidative stress contributes to vascular remodeling in hypertension [6,7]. We further examined the role of RAD in the superoxide production in arteries. Superoxide production was increased in the aorta and mesenteric artery, which was attenuated by RAD (Figure 6A). The increased NOX activity in the aorta and mesenteric artery of SHR was inhibited by RAD (Figure 6B). Moreover, the upregulated NOX1, NOX2 and NOX4 expressions in the aorta and mesenteric artery of SHR were attenuated by (Figure 7A,B). These results indicate that RAD attenuates oxidative stress in the arteries of SHR.

### 3.7. Vascular Remodeling

**Figure 8:**
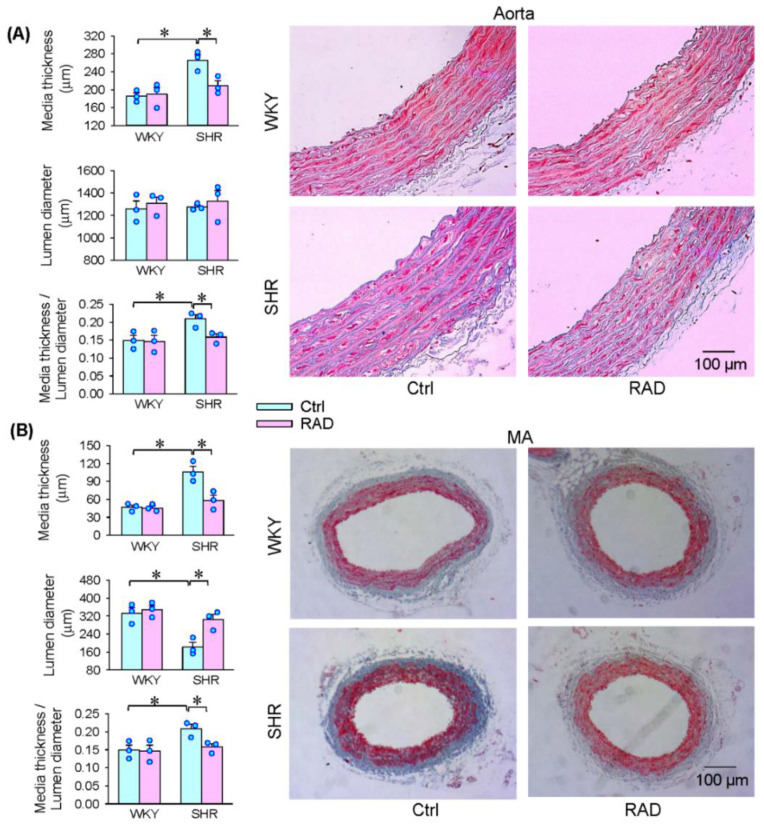
Effects of RAD on vascular remodeling of the aorta and mesenteric artery (MA) in WKY and SHR. Bar graphs show the Masson’s staining analysis for media thickness, lumen diameter and their ratio of arteries. Representative images showing Masson’s staining of the aorta (**A**) and MA (**B**). Values are mean ± SE. Two-way ANOVA followed by Bonferroni’s post hoc analysis were used for comparisons. * *p* < 0.05. *n* = 3 for each group.

The aorta and mesenteric artery were, respectively, used as a candidate of the large and small arteries. The media thickness and the ratio of media thickness to lumen diameter were increased in the aorta of SHR, which were attenuated by RAD. However, RAD had no significant effect on the lumen diameter in the aorta of SHR (Figure 8A). In the mesenteric artery, the media thickness and the media thickness/lumen diameter ratio were increased, but the lumen diameter was reduced in SHR, which was abolished by RAD (Figure 8B). These findings provide evidence that RAD attenuates vascular remodeling in SHR. It is worth noting that RAD had no significant effects on sympathetic activity, blood pressure, heart rate, plasma NE level, HRV, superoxide production, NOX activity and expression, and vascular remodeling in WKY (Figure 2, Figure 3, Figure 4, Figure 5, Figure 6, Figure 7 and Figure 8).

### 3.8. Serum and Urine Aldosterone, K^+^ and Na^+^ Levels

**Figure 9:**
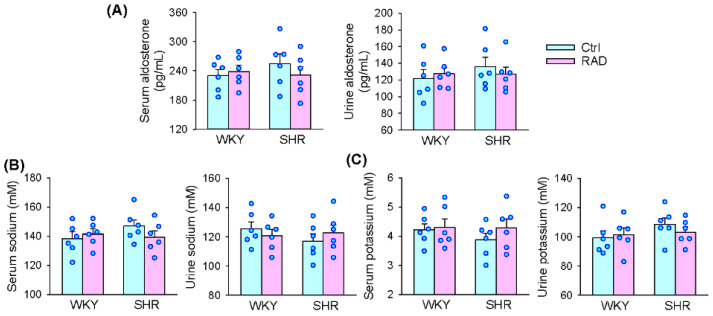
Effects of RAD on aldosterone, Na^+^ and K^+^ levels in serum and urine of WKY and SHR. (**A**) Serum and urine aldosterone levels. (**B**) Serum and urine Na^+^ levels. (**C**) Serum and urine K^+^ levels. Values are mean ± SE. Two-way ANOVA followed by Bonferroni’s post hoc analysis were used for comparisons. No significant difference among them. *n* = 6 for each group.

The measurements were performed in WKY and SHR aged 12 weeks, 3 weeks after RAD or sham intervention. There were no significant differences in the serum and urine aldosterone, K^+^ and Na^+^ levels between WKY and SHR. RAD had no significant effects on the aldosterone, K^+^ and Na^+^ levels in both WKY and SHR (Figure 9A–C).

## 4. Discussion

Catheter-based renal denervation is widely used as a therapy for resistant hypertension, which destroys both afferent and efferent renal fibers [23,24]. Capsaicin is known to stimulate afferents and induces excitation at low concentration, but capsaicin at high concentration (about 33 mM) causes transient excitation followed by persistent denervation [29,42]. This study shows the roles of selective ablation of renal afferent fibers without interrupting renal efferent activity in SHR. The primary novel findings are that RAD caused by high concentration of capsaicin attenuates sympathetic activation, superoxide signaling or oxidative stress in both the PVN and arteries, hypertension, and vascular remodeling in SHR. Selective removal of renal afferent innervation may be a therapeutic strategy for hypertension.

Elevated renal afferent activity increases sympathetic activity, and the excitatory reflex is enhanced in SHR, especially in the early stage of the hypertension [20], suggesting that selective removal of renal afferents may play beneficial roles in attenuating hypertension. However, surgical renal denervation in animals or catheter-based renal nerve ablation in humans is a nonselective method, which disrupts both afferent and efferent renal fibers [23,24]. Several methods have been tried for selective ablation of afferents. For example, systemic administration of capsaicin, a transient receptor potential receptor 1 (TRPV1) agonist, in neonatal rat pups does ablate renal afferents, but results in degeneration of all the TRPV1-positive sensory fibers throughout the body [43,44,45]. Surgical sectioning of dorsal roots of the spinal cord (dorsal rhizotomy) at T_9_-L_1_ does remove most renal afferent fibers to the spinal cord, but disrupts all cutaneous, somatic and visceral afferents at the levels [46,47]. Both methods specifically abolish sensory fibers, but are not selective for renal afferents. The ablation of other sensory afferents may cause severe dysfunction or side effects. It is well documented that capsaicin ablates unmyelinated C-fibers [48]. Topical application of capsaicin to the adrenal gland of rats induces selective denervation of adrenal afferents [49]. It is known that the majority of renal afferent fibers are capsaicin-sensitive unmyelinated fibers [50,51], and TRPV1 receptors are localized along the axons and terminals of renal sensory fibers [52]. The method used in the present study selectively ablates renal afferents without interrupting renal sympathetic output, which has been well documented in previous studies [25,26,29]. The efficiency of renal afferent denervation caused by capsaicin treatment was confirmed by the reduction of CGRP expressions in the kidney in the present study. Importantly, we provide functional evidence that the capsaicin treatment had no significant damage effect on renal sympathetic activity.

Excessive sympathetic activity is crucial in the pathogeneses of hypertension [12]. Input from the kidney to the brain increases sympathetic activity and blood pressure via causing the ERR in WKY and SHR [16]. In this study, selectively abolishing the renal afferent activity attenuates sympathetic activity, which attributes to the abrogation of the ERR at the renal afferent level. Superoxide in the PVN is closely related to the integration of cardiovascular activity and sympathetic output, and increased superoxide production in the PVN is mainly responsible for sympathetic activation in hypertension [9]. Reducing superoxide levels in the PVN attenuates sympathetic activity and hypertension in hypertensive rats [53,54]. ERR-induced by activation of renal afferents is mediated by the AT_1_ receptors-superoxide pathway in the PVN [17]. RAD reduced NOX2/4 expressions and NOX activity as well as superoxide production, which is at least partially responsible for the inhibition of excessive sympathetic activity in SHR.

**Figure 10:**
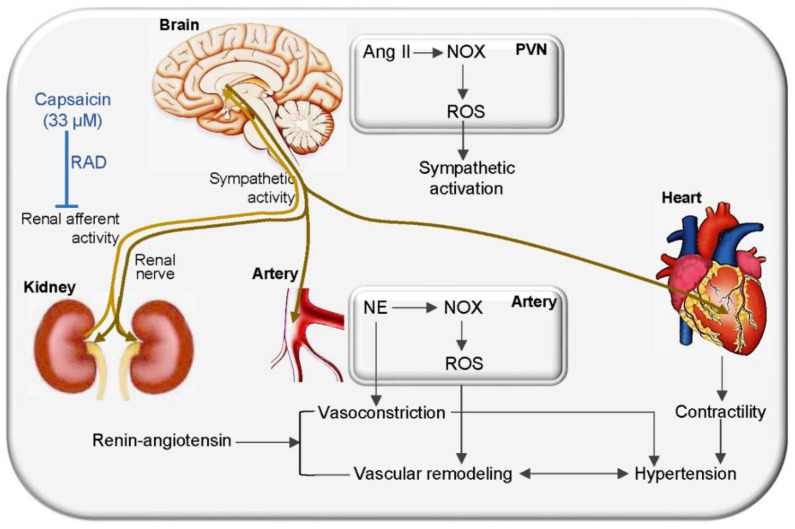
Schematic diagram showing the effects and mechanisms of renal afferent denervation (RAD) on vascular remodeling and hypertension in SHR.

Vascular oxidative stress contributes to vascular remodeling and hypertension [6,7]. RAD reduced NOX-related superoxide production in the aorta and mesenteric artery, which plays beneficial roles in attenuating vascular oxidative stress. It is known that increased NE induces oxidative stress [55,56]. Excessive sympathetic activity causes not only more NE release in the arteries, but a raise in the plasma NE level in SHR, which promotes vascular oxidative stress. Moreover, increased sympathetic activity and NE release promote hypertension, which further aggravates vascular oxidative stress. The role of RAD in attenuating vascular oxidative stress may be attributed to RAD-caused inhibition in sympathetic activity and NE release. The major role of selective RAD in attenuating vascular remodeling in SHR attributes to the reduced sympathetic activity, vascular oxidative stress and hypertension (Figure 10). On the other hand, sympathetic over-activity promotes renin release and Ang II generation [57]. Selective RAD attenuated sympathetic activity, which may have a beneficial effect in attenuating the activity of the renin–angiotensin system.

Device-based therapies such as catheter-based renal sympathetic denervation have been widely used to treat resistant hypertension, chronic kidney disease and chronic heart failure [23]. The technique interrupts the activity of both afferent and efferent renal nerves. In the present study, selective ablation of renal afferents plays beneficial roles in attenuated sympathetic activation, central and vascular oxidative stress, vascular remodeling, and hypertension in SHR, but no significant effects were observed in these aspects in WKY. The findings suggest that selective RAD may be an effective method for treating hypertension with less disadvantage in interfering physiological regulatory function, especially renal function. It is interesting that selective RAD had no significant effects in WKY. This finding suggests that renal afferent activity in physiological conditions is not important in the control of vascular oxidative stress and remodeling. It is speculated that selective RAD may cause some mild direct effects, but may be compensated by regulatory mechanisms to maintain homeostasis. However, whether there are long-term effects of RAD in normal rats needs further investigation.

Previous studies showed that RAD in rats with unilateral ischemic kidney reduced ROS production and renal dysfunction in the kidney [23,25], and that RAD attenuates proteinuria and renal fibrosis in rats with unilateral 5/6 nephrectomy [26]. However, it is unknown whether selective RAD had effects on blood and urine K^+^, Na^+^, and aldosterone levels. Previous studies showed that there were no significant increases in the blood urea nitrogen (BUN) and creatinine levels in SHR aged about 12 weeks [58,59,60]. There were no significant differences in the blood and urine Na^+^, K^+^, and aldosterone levels between WKY and SHR at the early stage of hypertension [61,62,63,64], which were further confirmed in the present study. Moreover, RAD had no significant effects on blood and urine Na^+^, K^+^, and aldosterone levels in both WKY and SHR. A possible explanation is that the SHR at this age are in the early stage of hypertension, without obvious renal dysfunction. However, renal dysfunction or injury certainly exist in the late stage of hypertension. Therefore, the long-term effects of the selective RAD or the effects of the selective RAD in older SHR deserves further investigation. A limitation in the present study was that blood pressure was measured with a computerized tail-cuff system, which is easily affected by the process of the measurements. Moreover, the DBP values obtained with this method was not very reliable.

## 5. Conclusions

Selective ablation of renal afferents attenuates sympathetic activity, vascular oxidative stress, vascular remodeling and hypertension in SHR. The abolished afferent activity from the kidney to the PVN reduces superoxide-mediated signaling in the PVN, and then causes the attenuation of sympathetic activity in SHR. The reduced sympathetic activity partially contributes to the attenuation of vascular oxidative stress and remodeling in hypertension.

## Data Availability

Data is contained within the article.

## References

[1] Garcia-Redondo A.B., Aguado A., Briones A.M., Salaices M. (2016). NADPH oxidases and vascular remodeling in cardiovascular diseases. Pharmacol. Res..

[2] La M.S., Lopez-Sanz L., Bernal S., Jimenez-Castilla L., Prieto I., Morelli G., Gomez-Guerrero C., Marasco D. (2020). Antioxidant effects of PS5, a peptidomimetic of suppressor of cytokine signaling 1, in experimental atherosclerosis. Antioxidants.

[3] Ho C.C., Chen Y.C., Tsai M.H., Tsai H.T., Weng C.Y., Yet S.F., Lin P. (2021). Ambient particulate matter induces vascular smooth muscle cell phenotypic changes via NOX1/ROS/NF-kB dependent and independent pathways: Protective effects of polyphenols. Antioxidants.

[4] Wu N., Ye C., Zheng F., Wan G.W., Wu L.L., Chen Q., Li Y.H., Kang Y.M., Zhu G.Q. (2020). MiR155-5p inhibits cell migration and oxidative stress in vascular smooth muscle cells of spontaneously hypertensive rats. Antioxidants.

[5] Obradovic M., Essack M., Zafirovic S., Sudar-Milovanovic E., Bajic V.P., Van N.C., Trpkovic A., Stanimirovic J., Bajic V.B., Isenovic E.R. (2020). Redox control of vascular biology. Biofactors.

[6] Touyz R.M., Rios F.J., ves-Lopes R., Neves K.B., Camargo L.L., Montezano A.C. (2020). Oxidative Stress: A Unifying Paradigm in Hypertension. Can. J. Cardiol..

[7] Krzeminska J., Wronka M., Mfynarska E., Franczyk B., Rysz J. (2022). Arterial Hypertension-Oxidative Stress and Inflammation. Antioxidants.

[8] Zhou B., Wu L.L., Zheng F., Wu N., Chen A.D., Zhou H., Chen J.Y., Chen Q., Li Y.H., Kang Y.M. (2021). miR-31-5p Promotes Oxidative Stress and Vascular Smooth Muscle Cell Migration in Spontaneously Hypertensive Rats via Inhibiting FNDC5 Expression. Biomedicines.

[9] Chen W.W., Xiong X.Q., Chen Q., Li Y.H., Kang Y.M., Zhu G.Q. (2015). Cardiac sympathetic afferent reflex and its implications for sympathetic activation in chronic heart failure and hypertension. Acta Physiol..

[10] Cheng Z.J., Wang R., Chen Q.H. (2019). Autonomic rregulation of the cardiovascular system: Diseases, treatments, and novel approaches. Neurosci. Bull..

[11] Ye C., Zheng F., Nan W., Zhu G.Q., Li X.Z. (2022). Extracellular vesicles in vascular remodeling. Acta Pharmacol. Sin..

[12] Grassi G., Mark A., Esler M. (2015). The sympathetic nervous system alterations in human hypertension. Circ. Res..

[13] De Lalio L.J., Sved A.F., Stocker S.D. (2020). Sympathetic Nervous System Contributions to Hypertension: Updates and Therapeutic Relevance. Can. J. Cardiol..

[14] Osborn J.W., Tyshynsky R., Vulchanova L. (2021). Function of Renal Nerves in Kidney Physiology and Pathophysiology. Annu. Rev. Physiol..

[15] Kaur J., Young B.E., Fadel P.J. (2017). Sympathetic overactivity in chronic kidney disease: Consequences and mechanisms. Int. J. Mol. Sci..

[16] Ye C., Qiu Y., Zhang F., Chen A.D., Zhou H., Wang J.J., Chen Q., Li Y.H., Kang Y.M., Zhu G.Q. (2020). Chemical stimulation of renal tissue induces sympathetic activation and pressor response via hypothalamic paraventricular nucleus. Neurosci. Bull..

[17] Qiu Y., Zheng F., Ye C., Chen A.D., Wang J.J., Chen Q., Li Y.H., Kang Y.M., Zhu G.Q. (2020). Angiotensin type 1 receptors and superoxide anion production in hypothalamic paraventricular nucleus contribute to capsaicin-induced excitatory renal reflex and sympathetic activation. Neurosci. Bull..

[18] Zheng F., Ye C., Wan G.W., Zhou B., Tong Y., Lei J.Z., Chen Q., Li Y.H., Kang Y.M., Zhu G.Q. (2020). Interleukin-1b in hypothalamic paraventricular nucleus mediates excitatory renal reflex. Pflugers Arch..

[19] Xu B., Zheng H., Liu X., Patel K.P. (2015). Activation of afferent renal nerves modulates RVLM-projecting PVN neurons. Am. J. Physiol. Heart Circ. Physiol..

[20] Ye C., Zheng F., Wang J.X., Wang X.L., Chen Q., Li Y.H., Kang Y.M., Zhu G.Q. (2021). Dysregulation of the excitatory renal reflex in the sympathetic activation of spontaneously hypertensive rat. Front. Physiol..

[21] Kopp U.C. (2015). Role of renal sensory nerves in physiological and pathophysiological conditions. Am. J. Physiol. Regul. Integr. Comp. Physiol..

[22] Milanez M.I.O., Veiga A.C., Martins B.S., Pontes R.B., Bergamaschi C.T., Campos R.R., Nishi E.E. (2020). Renal sensory activity regulates the g-aminobutyric acidergic inputs to the paraventricular nucleus of the hypothalamus in Goldblatt hypertension. Front. Physiol..

[23] Lauder L., Bohm M., Mahfoud F. (2021). The current status of renal denervation for the treatment of arterial hypertension. Prog. Cardiovasc. Dis..

[24] Weber M.A., Osborn J.W. (2021). Improved Understanding of Renal Nerve Anatomy: An Opportunity to Enhance Denervation Treatment of Hypertension. JACC Cardiovasc. Interv..

[25] Lopes N.R., Milanez M.I.O., Martins B.S., Veiga A.C., Ferreira G.R., Gomes G.N., Girardi A.C., Carvalho P.M., Nogueira F.N., Campos R.R. (2020). Afferent innervation of the ischemic kidney contributes to renal dysfunction in renovascular hypertensive rats. Pflugers Arch..

[26] Veiga A.C., Milanez M.I.O., Ferreira G.R., Lopes N.R., Santos C.P., De A.K., Garcia M.L., Oyama L.M., Gomes G.N., Nogueira F.N. (2020). Selective afferent renal denervation mitigates renal and splanchnic sympathetic nerve overactivity and renal function in chronic kidney disease-induced hypertension. J. Hypertens.

[27] Graham D., McBride M.W., Brain N.J., Dominiczak A.F. (2005). Congenic/consomic models of hypertension. Methods Mol Med.

[28] Bell D., Kelso E.J., Argent C.C., Lee G.R., Allen A.R., McDermott B.J. (2004). Temporal characteristics of cardiomyocyte hypertrophy in the spontaneously hypertensive rat. Cardiovasc. Pathol..

[29] Foss J.D., Wainford R.D., Engeland W.C., Fink G.D., Osborn J.W. (2015). A novel method of selective ablation of afferent renal nerves by periaxonal application of capsaicin. Am. J. Physiol. Regul. Integr. Comp. Physiol..

[30] Kondo H., Kondo M., Hayashi K., Kusafuka S., Hamamura K., Tanaka K., Kodama D., Hirai T., Sato T., Ariji Y. (2021). Orthodontic tooth movement-activated sensory neurons contribute to enhancing osteoclast activity and tooth movement through sympathetic nervous signalling. Eur. J. Orthod..

[31] Giancola F., Gentilini F., Romagnoli N., Spadari A., Turba M.E., Giunta M., Sadeghinezhad J., Sorteni C., Chiocchetti R. (2016). Extrinsic innervation of ileum and pelvic flexure of foals with ileocolonic aganglionosis. Cell Tissue Res..

[32] Xiong X.Q., Chen W.W., Zhu G.Q. (2014). Adipose afferent reflex: Sympathetic activation and obesity hypertension. Acta Physiol..

[33] Shi Z., Chen W.W., Xiong X.Q., Han Y., Zhou Y.B., Zhang F., Gao X.Y., Zhu G.Q. (2012). Sympathetic activation by chemical stimulation of white adipose tissues in rats. J. Appl. Physiol..

[34] Xiong X.Q., Chen W.W., Han Y., Zhou Y.B., Zhang F., Gao X.Y., Zhu G.Q. (2012). Enhanced adipose afferent reflex contributes to sympathetic activation in diet-induced obesity hypertension. Hypertension.

[35] Wu L.L., Bo J.H., Zheng F., Zhang F., Chen Q., Li Y.H., Kang Y.M., Zhu G.Q. (2021). Salusin-b in intermediate dorsal motor nucleus of the vagus regulates sympathetic-parasympathetic balance and blood pressure. Biomedicines.

[36] Task Force of the European Society of Cardiology the North American Society of Pacing Electrophysiology (1996). Heart rate variability: Standards of measurement, physiological interpretation and clinical use. Task Force of the European Society of Cardiology and the North American Society of Pacing and Electrophysiology. Circulation.

[37] Garde A.H., Laursen B., Jorgensen A.H., Jensen B.R. (2002). Effects of mental and physical demands on heart rate variability during computer work. Eur. J. Appl. Physiol..

[38] Wu N., Zheng F., Li N., Han Y., Xiong X.Q., Wang J.J., Chen Q., Li Y.H., Zhu G.Q., Zhou Y.B. (2021). RND3 attenuates oxidative stress and vascular remodeling in spontaneously hypertensive rat via inhibiting ROCK1 signaling. Redox Biol..

[39] Sun H.J., Ren X.S., Xiong X.Q., Chen Y.Z., Zhao M.X., Wang J.J., Zhou Y.B., Han Y., Chen Q., Li Y.H. (2017). NLRP3 inflammasome activation contributes to VSMC phenotypic transformation and proliferation in hypertension. Cell Death Dis..

[40] Ren X.S., Tong Y., Qiu Y., Ye C., Wu N., Xiong X.Q., Wang J.J., Han Y., Zhou Y.B., Zhang F. (2020). MiR155-5p in adventitial fibroblasts-derived extracellular vesicles inhibits vascular smooth muscle cell proliferation via suppressing angiotensin-converting enzyme expression. J. Extracell Vesicles.

[41] Liu T.Y., Shi C.X., Gao R., Sun H.J., Xiong X.Q., Ding L., Chen Q., Li Y.H., Wang J.J., Kang Y.M. (2015). Irisin inhibits hepatic gluconeogenesis and increases glycogen synthesis via the PI3K/Akt pathway in type 2 diabetic mice and hepatocytes. Clin. Sci..

[42] Fitzgerald M. (1983). Capsaicin and sensory neurones—A review. Pain.

[43] Wang D.H., Li J., Qiu J. (1998). Salt-sensitive hypertension induced by sensory denervation: Introduction of a new model. Hypertension.

[44] Wang D.H., Wu W., Lookingland K.J. (2001). Degeneration of capsaicin-sensitive sensory nerves leads to increased salt sensitivity through enhancement of sympathoexcitatory response. Hypertension.

[45] Wang Y., Chen A.F., Wang D.H. (2005). ET(A) receptor blockade prevents renal dysfunction in salt-sensitive hypertension induced by sensory denervation. Am. J. Physiol. Heart Circ. Physiol..

[46] Kopp U.C., Cicha M.Z., Smith L.A. (2003). Dietary sodium loading increases arterial pressure in afferent renal-denervated rats. Hypertension.

[47] Kopp U.C., Jones S.Y., DiBona G.F. (2008). Afferent renal denervation impairs baroreflex control of efferent renal sympathetic nerve activity. Am. J. Physiol. Regul. Integr. Comp. Physiol..

[48] Holzer P. (1991). Capsaicin: Cellular targets, mechanisms of action, and selectivity for thin sensory neurons. Pharmacol Rev.

[49] Ulrich-Lai Y.M., Fraticelli A.I., Engeland W.C. (2003). Capsaicin-sensitive nerve fibers: A potential extra-ACTH mechanism participating in adrenal regeneration in rats. Microsc Res Tech.

[50] Wang H., Wang D.H., Galligan J.J. (2010). P2Y2 receptors mediate ATP-induced resensitization of TRPV1 expressed by kidney projecting sensory neurons. Am. J. Physiol. Regul. Integr. Comp. Physiol..

[51] Knuepfer M.M., Schramm L.P. (1987). The conduction velocities and spinal projections of single renal afferent fibers in the rat. Brain Res..

[52] Szallasi A., Blumberg P.M. (1999). Vanilloid (Capsaicin) receptors and mechanisms. Pharmacol. Rev..

[53] Sun H.J., Zhang L.L., Fan Z.D., Chen D., Zhang L., Gao X.Y., Kang Y.M., Zhu G.Q. (2014). Superoxide anions involved in sympathoexcitation and pressor effects of salusin-beta in paraventricular nucleus in hypertensive rats. Acta Physiol..

[54] Yuan N., Zhang F., Zhang L.L., Gao J., Zhou Y.B., Han Y., Zhu G.Q. (2013). SOD1 gene transfer into paraventricular nucleus attenuates hypertension and sympathetic activity in spontaneously hypertensive rats. Pflugers Arch..

[55] Schraml E., Quan P., Stelzer I., Fuchs R., Skalicky M., Viidik A., Schauenstein K. (2007). Norepinephrine treatment and aging lead to systemic and intracellular oxidative stress in rats. Exp. Gerontol..

[56] Deo S.H., Jenkins N.T., Padilla J., Parrish A.R., Fadel P.J. (2013). Norepinephrine increases NADPH oxidase-derived superoxide in human peripheral blood mononuclear cells via a-adrenergic receptors. Am. J. Physiol. Regul. Integr. Comp. Physiol..

[57] Hering L., Rahman M., Potthoff S.A., Rump L.C., Stegbauer J. (2020). Role of a-adrenoceptors in hypertension: Focus on renal sympathetic neurotransmitter release, inflammation, and sodium homeostasis. Front. Physiol..

[58] Kato T., Mizuguchi N., Ito A. (2015). Blood pressure, renal biochemical parameters and histopathology in an original rat model of essential hypertension (SHRSP/Kpo strain). Biomed. Res..

[59] Qiu L., Zhang J., Yang Y., Zhang H., Lee F.F., He Q., Huang C., Huang L., Qian L., Luo J. (2022). In vivo assessment of hypertensive nephrosclerosis using ultrasound localization microscopy. Med. Phys..

[60] Naruse M., Tanabe A., Naruse K., Adachi C., Yoshimoto T., Seki T., Takagi S., Imaki T., Watanabe T., Takano K. (2000). Hemodynamic and biochemical effects of endothelin-A- and -B-receptor antagonist TAK-044 in stroke-prone spontaneously hypertensive rats. J. Cardiovasc. Pharmacol..

[61] Wang X., Zhu Y., Wang S., Wang Z., Sun H., He Y., Yao W. (2020). Effects of eplerenone on cerebral aldosterone levels and brain lesions in spontaneously hypertensive rats. Clin. Exp. Hypertens.

[62] Qin F., Li J., Dai Y.F., Zhong X.G., Pan Y.J. (2022). Renal denervation inhibits the renin-angiotensin-aldosterone system in spontaneously hypertensive rats. Clin. Exp. Hypertens.

[63] Naruse M., Tanabe A., Sato A., Takagi S., Tsuchiya K., Imaki T., Takano K. (2002). Aldosterone breakthrough during angiotensin II receptor antagonist therapy in stroke-prone spontaneously hypertensive rats. Hypertension.

[64] Pereira-Derderian D.T., Vendramini R.C., Menani J.V., De Luca L.A. (2010). Water deprivation-induced sodium appetite and differential expression of encephalic c-Fos immunoreactivity in the spontaneously hypertensive rat. Am. J. Physiol. Regul. Integr. Comp. Physiol..

